# Tumor Necrosis Factor-Alpha −308 G>A Polymorphism, Adherence to Mediterranean Diet, and Risk of Overweight/Obesity in Young Women

**DOI:** 10.1155/2014/742620

**Published:** 2014-06-17

**Authors:** Martina Barchitta, Annalisa Quattrocchi, Veronica Adornetto, Anna Elisa Marchese, Antonella Agodi

**Affiliations:** ^1^Department of Anatomy, Biology and Genetics, Forensic Medicine, Neuroscience, Diagnostic Pathology, Hygiene and Public Health, University of Catania, Via S. Sofia 87, 95123 Catania, Italy; ^2^UOC Laboratorio Analisi 1, P.O. “Vittorio Emanuele”, Via Plebiscito 628, 95100 Catania, Italy

## Abstract

The present study was conducted in order to (i) characterize the adherence to the Mediterranean diet (MD) pattern and fatty acids (FAs) intakes and (ii) explore interactions between *TNFA* −308 G>A polymorphism and adherence to MD and FAs intakes, respectively, on overweight/obesity risk. From 2010 to 2013, 380 healthy women were enrolled, and MD score (MDS) and FAs intakes were evaluated by a Food Frequencies Questionnaire in relation to nutritional status. *TNFA* −308 G/A polymorphism was characterized using PCR-RFLP. A total of 32.6% of women were overweight or obese. Lower mean MDS values were more observed in the younger age group than in the older age group (3.60 versus 4.45). The risk of being overweight/obese was 3.5-fold increased due to poor adherence to MD and was about twofold increased in less educated women. Furthermore, younger age was associated with poor adherence to MD. No evidence for an independent effect of the polymorphism on overweight/obesity risk was found. There was no evidence of biological interaction from the gene-diet interaction analyses. Young women, less educated and with poor adherence to MD, are a target group for the nutritional interventions that aimed to control the obesity risk, thus improving the adherence to MD and particularly the intake of unsaturated FAs.

## 1. Introduction

Obesity is a multifactorial disorder, reflecting complex interactions of genes and environment, as lifestyle [1], associated with a high risk of chronic diseases such as diabetes, cardiovascular disease, and certain cancers [2]. Obesity constitutes a major public health problem that, in current years, evolved into a worldwide epidemic [3]. A recent study conducted in the Diogenes (Diet, Obesity, and Genes Dietary Study in European countries) cohort [4] shows an increase in obesity prevalence since the 1990s and predicts a further increase in European populations of about 30% in 2015.

The Mediterranean diet (MD) has long been reported to be the optimal diet for preventing noncommunicable diseases and preserving good health. A meta-analysis confirms the significant and consistent protection provided by adherence to the MD in relation to the occurrence and mortality of major chronic degenerative diseases [5, 6].

Independently of energy and macronutrient quantity intakes, a better adherence to the MD is associated with lower obesity risk [7, 8]. As such, research interest over the past years has been focused on estimating adherence to the whole MD rather than analyzing the individual components of the dietary pattern in order to consider important interactions between components of the diet.

Lifestyle factors, including dietary components, such as fatty acids (FAs), interact with genetic variants to regulate the development and progression of obesity and its comorbidities, and these interactions may explain differences observed across populations [9].

A number of candidate genes have been implicated in the pathogenesis of obesity in humans, and screenings of those candidate regions as well as genome-wide scans have helped to identify single nucleotide polymorphisms (SNPs) that increase the risk of overweight or of obesity [10].

Obesity is a chronic low-grade inflammatory state, and elevated levels of tumor necrosis factor-alpha (TNF-*α*), a proinflammatory cytokine secreted in adipocytes, have been implicated in the development of obesity and insulin resistance; in fact, expression and circulating levels are increased with obesity and decreased with weight loss [11]. Several SNPs have been identified in the promoter region of* TNFA* gene. A meta-analysis by Sookoian et al. [12] described the impact of the best characterized* TNFA* polymorphism (−308 G>A; rs1800629) on the components of the metabolic syndrome and concluded that the −308 A variant was positively associated with obesity. The* TNFA*  −308 A allele has been associated with obesity, obesity-related insulin resistance, and altered serum lipid concentrations in some Caucasian populations [13, 14], but not all [15, 16]. In addition, in some populations, the* TNFA*  −308 G>A polymorphism changes the relationship between FAs intake and the risk of obesity [9, 17], but this interaction was not observed in other populations [18]. Furthermore, researches using* in vitro* and* in vivo* mouse models have shown that* TNFA* expression is differentially regulated by FAs, and these results appear to translate to human [19]. Although the molecular mechanisms by which FAs regulate adipokine production remain unclear, one proposed link between dietary FA and inflammation may be via the toll-like receptor 4 (TLR4) pathway [20]. TLR4 is expressed in adipose tissue and has been shown to be activated by SFA, inducing inflammatory cytokine production and signalling. This results in a localized inflammation in adipose tissue that propagates an overall systemic inflammation [21, 22].

The main aim of the present study was to assess the risk of overweight/obesity in a Sicilian population of healthy women and to define control strategies and targets. Particularly, the specific purposes were (i) to characterize adherence to MD pattern and FAs intakes and (ii) to explore interactions between* TNFA*  −308 G>A polymorphism and adherence to MD pattern and FAs intakes, respectively, on overweight/obesity risk.

## 2. Materials and Methods

### 2.1. Study Population and Dietary Intake

During a three-year period, from 2010 to 2013, a total of 380 consecutive healthy women, referred to the Laboratory of the S. Bambino Hospital, Catania, Sicily, Italy, were prospectively enrolled in the present cross-sectional study. All women gave their informed consent to participate in the study. The study protocol was approved by the ethics committee of the involved institution and was performed according to the Declaration of Helsinki.

Data were collected by trained epidemiologists using a structured questionnaire to obtain information on demographic and lifestyle data, including smoking habits and obstetrical history.

Furthermore, education level was collected and divided into three categories: ≤8 (low), >8 and ≤13 (medium), and >13 (high) years of studies. Employment status was also recorded, and women were classified as employed, unemployed, student, and housewife. Body mass index (BMI) as kg/m^2^ was based on criteria from the World Health Organization [23]. Prepregnancy BMI was based on self-reported prepregnancy weight.

Adherence to MD and FAs intakes, during the past month, was estimated by a semiquantitative 153-item Food Frequencies Questionnaire (FFQ), previously validated [24–26]. For each of the food items, women were asked to report their frequency of consumption and portion size. The table of alimentary composition of the US Department of Agriculture, which had been modified to accommodate the particularities of the Italian diet, was used to determine FAs intakes. FAs intakes were evaluated both as average intakes in grams per day (g/day) and as percentage of energy (%E) using the “nutrient density” method [27]. Total daily energy intake was calculated as Kcal of energy provided by macronutrients (total proteins, carbohydrates, and lipids) and alcohol.

Adherence to the MD pattern was assessed using the Mediterranean diet score (MDS) [28]. Women were classified as with greater adherence to MD if MDS was >90th percentile of MDS distribution (i.e., >6) and as with poor adherence to MD (i.e., ≤90th percentile: ≤6).

### 2.2. Analysis of* TNFA*  −308 G>A Polymorphism

Fasting venous blood samples were collected from each enrolled woman in EDTA-containing tubes, and aliquots were stored at −80°C until analysis.

Genomic DNAs were extracted from whole blood using the Illustra blood genomic Prep Mini Spin Kit (GE Healthcare) according to the manufacturer's protocol and stored at −20°C. Subjects were genotyped for the* TNFA*  −308 G>A polymorphism using the PCR-RFLP method, as described previously [29]. Electrophoresis of the digested PCR products was performed on a 5% NuSieve agarose gel (Lonza, ME, USA). Gels were stained with GelRed (Biotium, Inc., Hayward, CA, USA) in order to visualize the DNA fragments.

### 2.3. Statistical Analyses

Statistical analyses were performed using the SPSS software (Version 14.0, SPSS, Chicago, IL). The *χ* ^2^ test was used for the statistical comparison of proportions. Continuous variables were tested using Student's *t*-test and one-way ANOVA. Genotype frequencies were calculated by determining the percentage of individuals carrying the different genotypes. In the analysis, the number of homozygote wild-type individuals (GG) was compared to the number of heterozygotes and homozygote mutant individuals (AG/AA). Furthermore, to ascertain if population sample was in Hardy-Weinberg equilibrium for the polymorphisms, a *χ* ^2^ test was performed for the overall population.

Statistical significance of relationship between adherence to MD, overweight/obesity, and risk factors was determined using the *χ* ^2^ test, and the strength of associations was estimated by calculating the odds ratios (ORs) and 95% confidence intervals (95% CIs). Statistical significance was established at a *P* value of 0.05.

Gene-environment interaction analyses were conducted in order to evaluate the potential interaction between* TNFA*  −308 G>A polymorphism and adherence to MD pattern or FAs intakes, respectively, on overweight/obesity risk, using as a reference group the homozygous wild-type (GG) women who had not been exposed to dietary factors (i.e., those with MDS > 6 or with FAs intakes above the 75th percentile of the unsaturated FAs intakes distribution or below or equal to the 75th percentile of the saturated FAs intakes distribution, resp.). Furthermore, biological interaction analyses, using departure from additivity, were performed using the synergy index proposed by Rothman et al. [29, 30] with adjusted ORs and their 95% CIs measured from logistic regression analysis.

## 3. Results

### 3.1. Population Characteristics and Distribution of* TNFA*  −308 G>A Polymorphism

During the study period, a total of 380 women (mean age 28.7 years) of a southern European Mediterranean population were enrolled. The main characteristics of the women are shown in Table 1. Particularly, a total of 32.6% of women were overweight or obese.

The distribution of* TNFA*  −308 G>A polymorphism is shown in Table 1: the most frequent genotype was the wild-type GG (80.5%). The G allele frequency was 89.6%. Genotype frequencies follow the Hardy-Weinberg equilibrium expectations (*P* = 0.27).

### 3.2. Dietary Assessment

Mean energy and FAs intakes (g/day) of underweight/normal weight women and of those who are overweight/obese are reported in Table 2. Except for unsaturated/saturated FA ratio, the mean values of FAs intakes (unsaturated and saturated FAs) and the mean energy intake were statistically significantly higher in underweight/normal weight women than in overweight/obese women. However, considering the mean FAs intakes as daily %E, differences between the two groups were not statistically significant (data not shown).

According to MDS (Table 1), women reported a poor adherence to MD (median value of MDS equal to 4), and only 8.2% of women were classified as with greater adherence to MD (MDS > 90th percentile of MDS distribution, i.e., >6).

A significantly higher proportion of women with poor adherence to MD (34.4%) were more overweight/obese than those with greater adherence (12.9%; *P* = 0.015). Therefore, the risk of being overweight/obese due to poor adherence to MD was 3.5-fold increased (OR: 3.54, 95% CI: 1.21–10.34).

Following the percentile distribution, the population was divided into four age groups, and mean MDS values were compared between groups. A significant increase of mean MDS values was observed from 3.60 in the age group of 13–22 years to 4.04 in the age group of 23–33 years, to 4.24 in the age group of 34–41 years, and to 4.45 in the age group of 42–85 years (one-way ANOVA, *P* = 0.004). Therefore, the risk of poor adherence to MD was 2.2-fold increased in younger women (i.e., age ≤ 27 years median value).

Education was positively associated with adherence to MD; that is, less-educated women showed a lower adherence to MD, although this association was not statistically significant (data not shown).

A significant association between education and overweight/obesity was observed: 39.6% of women in lower (<=8), 26.1% in medium (>8 and ≤13), and 21.6% in highly (>13 years of school) educated groups were overweight or obese (*P* for trend = 0.003). The risk of being overweight/obese due to low-medium education was 1.94-fold increased (OR: 1.94, 95% CI: 1.25–3.02). Considering employment status, no statistically significant association was shown with adherence to MD; instead, 40.7% of housewives, 30.0% of the employed, 21.2% of the unemployed, and 19.6% of the students were overweight or obese (*P* for trend = 0.042).

Comparisons of mean energy and FAs intakes (g/day) between women with greater adherence to MD and those with poor adherence to MD are reported in Table 3. Except for some unsaturated FAs, that is, arachidonic acid and docosahexaenoic acid, for total saturated FA and palmitic acid, the mean FAs intakes and the mean energy intake were statistically significantly higher in women with greater adherence to MD than in women with poor adherence. Furthermore, considering the mean FAs intakes as daily %E, only the mean saturated FAs intakes were statistically significantly higher in women with poor adherence to MD than in the others (data not shown).

### 3.3. Gene-Environment Interactions

A total of 37.0% of carriers of the* TNFA*  −308 A allele (AA or GA genotypes) and a total of 31.6% of carriers of the* TNFA*  −308 GG genotype were overweight/obese, and this difference was not statistically significant (*P* = 0.383).

Results of the interaction analysis between* TNFA*  −308 G>A genotypes and FAs intakes in relation to obesity risk are reported in Tables 4 and 5. From our analysis, there was no evidence of gene-FA intakes interaction and of gene-MD adherence (data not shown).

## 4. Discussion

The present study was conducted in a Mediterranean population with a poor adherence to MD (median value of MDS equal to 4) and a high prevalence of overweight and obesity (32.6%), as recently reported among Italian and Sicilian adult women (33.8%) [26, 31]. Notably, in our population, the risk of being overweight/obese was 3.5-fold increased due to poor adherence to MD and was about twofold increased in less educated women. Furthermore, younger age was associated with poor adherence to MD, as shown in a recent study [32].

However, although the relationship between the MD and overweight/obesity is complex and important methodological differences (such as the methodology used to construct MD indices) and limitations in the studies make it difficult to compare results, the evidence points towards a possible role of the MD in preventing overweight/obesity and in protecting against weight gain, and, additionally, physiological mechanisms can explain this protective effect [33].

In recent years, southern European countries, which used to follow a traditional MD, have also been adopting a more Western-style diet, and a dramatic change in the sources of fat intake in the general population has been observed. This change mainly consists in replacing polyunsaturated or monounsaturated FAs, which have been considered as healthy lipids because they reduce the incidence of cardiovascular disease, with saturated FAs, recognized risk factors for cardiovascular disease [33].

However, in our study, women with greater adherence to MD consume significantly more unsaturated FAs (g/day) and less saturated FAs (daily %E) than women with poor adherence. Furthermore, unsaturated and saturated FAs intakes (g/day) were higher in underweight/normal weight women than in overweight/obese women, but, considering the mean FAs intakes as daily %E, differences between the two groups were not statistically significant (data not shown).

Some studies report that the MD pattern may be protective against the development of obesity through its high-fiber content and low energy density [27], but other studies have speculated that the high-fat content, particularly from olive oil, of the MD may promote excess energy intake and weight gain, and this may explain the high prevalence of overweight and obesity in Mediterranean countries [34]. In our population, mean energy intake was higher in women with greater adherence to MD than in women with poor adherence, and a similar association was previously reported [8, 35]. Nevertheless, it may be possible that this association is methodologically driven given that energy intake was not corrected for when constructing the MDS. Furthermore, mean energy intake was higher in underweight/normal weight women than in overweight/obese women, confirming that the root physiological cause of obesity is energy imbalance as a consequence of low physical activity and/or high energy intake, and several lifestyle factors may influence whether or not a person can maintain energy balance over the long term [7, 35, 36]. In fact, some studies support the theory that the problem of obesity in Mediterranean countries is likely to be related to limited physical activity in conjunction with excessive positive energy balance brought about by the westernization of their diet [37].

Lifestyle changes are the most important determinants of the rapid rise in the prevalence of obesity worldwide, and genetic factors are likely to modify the susceptibility to these changes [35]. The current lack of understanding of the numerous gene-gene and gene-environment interactions in obesity poses one of the major obstacles for the development of effective, preventive, and therapeutic intervention strategies [17]. It has been described that the A allele of the* TNFA*  −308 G>A polymorphism produces a twofold increase of* TNFA* transcription and subsequent increase in TNF-*α* production [38]. Furthermore, the polymorphism has been strongly associated with increased risk of different outcome in women such as spontaneous preterm birth [39].

Despite the fact that many studies show independent associations between the* TNFA* SNPs and obesity, only few studies have investigated diet-gene interactions. German Caucasian men and women with the* TNFA*  −308 A allele, who were in the highest tertile for intake of linoleic acid and arachidonic acid (%E), showed an increased obesity risk [40]. More recently, Joffe and colleagues reported that the odds of obesity for black South-African women with the* TNFA*  −308 A allele increased with total dietary fat intake (%E) [41]; however, this interaction was not observed in white South-African women [18].

Despite the biological plausibility of* TNFA*  −308 G>A polymorphism as risk modifiers of obesity, in the present study no evidence for an independent effect of the polymorphism on overweight/obesity risk was found. Also, there was no evidence of biological interaction from the gene-diet interaction analyses. These results are in keeping with previous findings in other populations [15, 16, 18], confirming the role of dietary factors, such as the FAs intake and the adherence to MD, in obesity risk irrespective of* TNFA*  −308 genotype. Additional SNPs within the* TNFA* gene, as well as SNPs in other genes involved in inflammation, may also be involved, and these should be investigated. Finally, other dietary factors, lifestyle, and environmental factors may modulate these associations and contribute to the different results observed.

## 5. Conclusions

A number of epidemiological studies have shown that greater adherence to the traditional MD is associated with a significant reduction in total mortality and death due to coronary heart disease and to cancer [37], could reduce overall cancer risk [2], and could provide a consistent protection for the occurrence of major chronic degenerative diseases [5]. Even though most of these chronic conditions are also associated with obesity, the link between MD and obesity is not clear.

The present research has certain limitations that need to be taken into account when considering the study and its contributions. Its cross-sectional design could limit the inference on the time sequence of the association between MD and nutritional status. In addition, selection bias, recall bias, and confounding might be present; indeed, women's diet can be especially difficult to assess, as women tend to underreport their intakes more often than men and are more likely to do so if they are overweight or obese [42], and this phenomenon will bias diet-disease relationships. Further, in our study, physical activity was not determined. Furthermore, although our FFQ is validated, it may contain measurement errors. Additionally, our research was limited to the assessment of only one SNP from a single gene, and it is known that a number of other candidate genes have been implicated in the pathogenesis of obesity.

In conclusion, our study identifies young women, less educated, and with poor adherence to MD as a cause for concern and a target group for nutritional interventions that aimed to control the obesity risk, thus improving the adherence to MD and particularly the intake of unsaturated FAs.

## Figures and Tables

**Table 1:** Characteristics of study participants (*N* = 380).

	*N* (%)
Education (years of schooling)	
≤8 (low)	202 (53.3)
≤13 (medium)	126 (33.2)
>13 (high)	51 (13.5)
Employment status	
Employed	120 (31.9)
Unemployed	33 (8.8)
Student	46 (12.2)
Housewife	177 (47.1)
BMI	
Underweight	33 (8.7)
Normal weight	223 (58.7)
Overweight	78 (20.5)
Obese	46 (12.1)
Smoking	
Current smokers	90 (23.7)
Nonsmokers	244 (64.4)
Former smokers	45 (11.9)
Pregnancy status (yes)	203 (53.4)
MDS	
0–3 (≤25th percentile)	140 (36.8)
4–6 (>25th percentile–≤90th percentile)	209 (55.0)
7–9 (>90th percentile)	31 (8.2)
*TNFA * −308 G>A genotypes	
GG	306 (80.5)
GA	69 (18.2)
AA	5 (1.3)

BMI: body mass index; MDS: Mediterranean diet score.

**Table 2:** Fatty acids and energy intakes in underweight/normal weight and overweight/obese women^a^.

Nutrient intake (g/day)	Underweight/normal weight	Overweight/obese	*P* value^b^
	Mean ± SD	Mean ± SD	
Linoleic acid	17.07 ± 12.09	12.66 ± 8.05	**<0.001**
Arachidonic acid	0.18 ± 0.28	0.13 ± 0.06	**0.012**
*γ*-Linolenic acid	1.60 ± 1.10	1.20 ± 0.54	**<0.001**
*α*-Linolenic acid	1.60 ± 1.11	1.21 ± 0.53	**<0.001**
Eicosapentaenoic acid	0.13 ± 0.23	0.08 ± 0.07	**0.011**
Docosahexaenoic acid	0.30 ± 0.67	0.20 ± 0.14	**0.031**
Polyunsaturated fatty acids	18.43 ± 10.10	14.50 ± 9.10	**<0.001**
Monounsaturated fatty acids	55.25 ± 25.24	47.41 ± 23.88	**0.004**
Total unsaturated fatty acids	73.68 ± 33.27	61.91 ± 31.62	**0.001**
Unsaturated/saturated fatty acids ratio	2.34 ± 0.69	2.34 ± 0.66	0.990
Saturated fatty acids	33.65 ± 18.13	27.33 ± 13.65	**<0.001**
Palmitic acid	19.35 ± 13.18	15.01 ± 7.12	**<0.001**
Energy intake (Kcal/day)	2272.0	1811.1	**<0.001**

^a^Statistically significant *P* values (*P* < 0.05) are indicated in bold font.

^b^Student's *t-*test for the comparison of means between underweight/normal weight women and overweight/obese women (two-sided *P* values).

**Table 3:** Fatty acids and energy intakes between women with greater adherence to MD and women with lower adherence to MD^a^.

Nutrient intake (g/day)	MDS ≤ 6	MDS > 6	*P* value^b^
	Mean ± SD	Mean ± SD	
Linoleic acid	15.24 ± 10.94	20.05 ± 12.39	**0.021**
Arachidonic acid	0.16 ± 0.24	0.17 ± 0.08	0.830
*γ*-Linolenic acid	1.44 ± 0.98	1.80 ± 0.75	**0.017**
*α*-Linolenic acid	1.44 ± 0.99	1.80 ± 0.67	**0.008**
Eicosapentaenoic acid	0.11 ± 0.20	0.16 ± 0.10	**0.021**
Docosahexaenoic acid	0.26 ± 0.58	0.34 ± 0.16	0.081
Polyunsaturated fatty acids	16.69 ± 9.53	22.35 ± 12.85	**0.002**
Monounsaturated fatty acids	51.36 ± 24.27	67.64 ± 28.95	**<0.001**
Total unsaturated fatty acids	68.05 ± 31.95	89.99 ± 39.90	**<0.001**
Unsaturated/saturated fatty acids ratio	2.32 ± 0.68	2.67 ± 0.62	**0.006**
Saturated fatty acids	31.26 ± 16.93	35.26 ± 18.18	0.210
Palmitic acid	17.74 ± 11.88	20.16 ± 9.65	0.197
Energy intake (Kcal/day)	2065.7 ± 897.5	2750 ± 1006.7	**<0.001**

^a^Statistically significant *P* values (*P* < 0.05) are indicated in bold font.

^b^Student's *t*-test for the comparison of means between MDS ≤ 6 and MDS > 6 (two-sided *P* values).

**Table 4:** *TNFA  *−308 G>A polymorphism and unsaturated fatty acids intake interactions on overweight/obesity risk^a,b^.

Fatty acids	A^c^ OR (95% CI)	B^d^ OR (95% CI)	A + B^e^ OR (95% CI)	Synergy index
Linoleic acid	2.68 (0.83–8.66)	3.61 (1.85–7.45)	4.27 (1.78–10.26)	0.76 (0.28–2.07)
Arachidonic acid	4.41 (1.53–12.72)	2.41 (1.25–4.64)	2.18 (0.94–5.05)	0.25 (0.06–1.00)
*γ*-Linolenic acid	3.06 (0.93–10.08)	3.47 (1.72–6.97)	3.80 (1.63–8.84)	0.62 (0.22–1.76)
*α*-Linolenic acid	3.98 (1.24–12.78)	4.20 (2.00–8.82)	4.40 (1.81–10.70)	0.55 (0.21–1.46)
Eicosapentaenoic acid	4.44 (1.43–13.73)	2.66 (1.35–5.23)	2.56 (1.10–5.94)	0.31 (0.09–1.10)
Docosahexaenoic acid	2.50 (0.85–7.32)	2.34 (1.21–4.54)	2.75 (1.19–6.34)	0.62 (0.18–2.12)
Polyunsaturated fatty acids	2.94 (0.90–9.56)	3.31 (1.64–6.68)	3.68 (1.57–8.63)	0.63 (0.22–1.84)
Monounsaturated fatty acids	2.58 (0.84–7.92)	2.18 (1.14–4.17)	2.48 (1.10–5.57)	0.54 (0.14–2.02)
Total unsaturated fatty acids	2.36 (0.77–7.20)	1.96 (1.03–3.72)	2.27 (1.02–5.07)	0.55 (0.13–2.29)
Unsaturated/saturated fatty acids ratio	0.86 (0.29–2.58)	0.71 (0.40–1.26)	1.13 (0.53–2.41)	—

^a^OR adjusted for age, adherence to MD, and education.

^b^Reference category: individuals carrying the homozygous wild-type genotype GG who had not been exposed to environmental factor.

^c^A: risk of developing overweight/obesity in carriers of *TNFA  *−308 A allele (individuals carrying the homozygous mutated genotype or the heterozygous genotype AA or AG, who had not been exposed to environmental factor-FAs intakes above the 75th percentile of the unsaturated FAs intakes distribution).

^d^B: risk of developing overweight/obesity in women exposed to environmental factor only (individuals carrying the homozygous wild-type genotype GG, who had been exposed to environmental factor-FAs intakes below or equal to the 75th percentile of the unsaturated FAs intakes distribution).

^e^A + B: risk of developing overweight/obesity in women exposed to both A and B (individuals carrying the homozygous mutated genotype or the heterozygous genotype AA or AG, who had been exposed to environmental factor-FAs intakes below or equal to the 75th percentile of the unsaturated FAs intakes distribution).

**Table 5:** *TNFA  *−308 G>A polymorphism and saturated fatty acids intake interactions on overweight/obesity risk^a,b^.

Fatty acids	A^c^ OR (95% CI)	B^d^ OR (95% CI)	A + B^e^ OR (95% CI)	Synergy index
Palmitic acid	0.94 (0.50–1.76)	0.26 (0.13–0.53)	1.34 (0.49–3.71)	—
Saturated fatty acids	1.11 (0.59–2.10)	0.29 (0.15–0.59)	0.90 (0.33–2.43)	—

^a^OR adjusted for age, adherence to MD, and education.

^b^Reference category: individuals carrying the homozygous wild-type genotype GG, who had not been exposed to environmental factor.

^c^A: risk of developing overweight/obesity in carriers of *TNFA  *−308 A allele (individuals carrying the homozygous mutated genotype or the heterozygous genotype AA or AG, who had not been exposed to environmental factor-FAs intakes below the 75th percentile of the saturated FAs intakes distribution).

^d^B: risk of developing overweight/obesity in women exposed to environmental factor only (individuals carrying the homozygous wild-type genotype GG, who had been exposed to environmental factor-FAs intakes above the 75th percentile of the saturated FAs intakes distribution).

^e^A + B: risk of developing overweight/obesity in women exposed to both A and B (individuals carrying the homozygous mutated genotype or the heterozygous genotype AA or AG, who had been exposed to environmental factor-FAs intakes above the 75th percentile of the saturated FAs intakes distribution).
